# Non-linear relationship between body mass index and self-rated health in older Korean adults: body image and sex considerations

**DOI:** 10.4178/epih.e2023061

**Published:** 2023-06-20

**Authors:** Seok-Joon Yoon, Jin-Gyu Jung, Soon-Ki Ahn, Jong-Sung Kim, Jang-Hee Hong

**Affiliations:** 1 Department of Family Medicine, Chungnam National University Hospital, Daejeon, Korea; 2 Department of Preventive Medicine, Chungnam National University Hospital, Daejeon, Korea; 3 Clinical Trial Center, Biomedical Research Institute, Chungnam National University Hospital, Daejeon, Korea

**Keywords:** Body mass index, Body image, Health, Aged

## Abstract

**OBJECTIVES:**

The purpose of this study was to investigate the association between body mass index (BMI) and self-rated health (SRH) in older adults aged over 65 years while examining the influence of self-perceived body image (SBI) and sex.

**METHODS:**

Raw data were obtained from the Korea Community Health Survey, which included BMI measurements of Koreans aged over 65 years (n=59,628). Non-linear relationships between BMI and SRH were analyzed separately for each sex using restricted cubic splines while controlling for SBI and other confounding variables.

**RESULTS:**

Men showed a reverse J-shaped association, while women showed a J-shaped association between BMI and poor SRH. However, including SBI in the model changed this association for men to an inverted U-shape showing a negative direction, with the highest risk of poor SRH observed in the underweight to overweight range. For women, a nearly linear positive relationship was observed. Regardless of BMI, those who perceived their weight as not “exactly the right weight” had a higher risk of poor SRH than those who perceived their weight as “exactly the right weight” in both men and women. Older men who thought they were much too fat or too thin had similar highest risks of poor SRH, whereas older women who thought they were too thin had the highest risk of poor SRH.

**CONCLUSIONS:**

The findings of this study emphasize the importance of considering sex and body image perceptions when assessing the relationship between BMI and SRH in older adults, especially in men.

## GRAPHICAL ABSTRACT

[Fig f4-epih-45-e2023061]

## INTRODUCTION

Self-rated health (SRH) has been widely used in social health studies since the 1950s, and its application in epidemiology and medical science has led to the recognition that it is associated with mortality [1]. SRH has been used in research in numerous countries and is widely addressed in areas such as risk prediction and clinical research [1]. In particular, SRH is known to be related to mortality prediction for older adults [2,3]. Several factors have been suggested to be associated with poor SRH, including stress, low education level, low social support, low social activity, activity restrictions due to various reasons, chronic disease, female sex, mobility difficulties, living alone, poor mental health, and obesity [4-6].

Obesity, as measured by body mass index (BMI), is related to major health burdens such as diabetes, hypertension, and cardiovascular disease [7]. Obesity contributes to a low quality of life and, in older women, is associated not only with depression but also with arthritis of the knee and hip joints [8,9]. Obesity in older adults is associated with a high risk of falls due to declining body function and insecurity about maintaining balance [10,11].

Numerous studies have investigated the relationship between obesity and SRH, with varying results. A study examining factors related to SRH in older adults in 11 European countries demonstrated that the relationship between obesity and SRH differed among countries [5]. A large-scale cohort study conducted in the United Kingdom suggested that obesity was associated with poor SRH in adults aged < 75 years [12]. In a cohort study conducted on older adults in Taiwan, baseline data indicated that poor SRH was more prevalent in obese individuals, but when baseline SRH was good and then worsened, SRH was not related to obesity [13]. Some reports have suggested that obesity is related to poor SRH in individuals under 50 years of age, but in adults over 50 years of age, the two are unrelated [14]. A study conducted in Korea showed that in both men and women over 65 years of age, obesity was not related to poor SRH [15].

In studies that did not limit subjects to older adults, perceived weight status played a significant role. In a study of Canadian adults [16], obese subjects who believed themselves to be of normal weight exhibited SRH similar to non-obese subjects. Other studies conducted in Korea also showed that both men and women who perceived themselves to be of normal weight, despite being obese, reported “not bad” SRH [17]. A study conducted in China showed that increased weight, not BMI, was associated with poor SRH [18].

Given these results and the tendency of many older adults who are overweight or obese to underestimate their weight [19], it is important to consider self-perceived body image (SBI) when examining the relationship between BMI and SRH in older adults. However, little research has investigated the relationships among BMI, SBI, and SRH. Therefore, this study aims to determine how BMI and SBI are related to poor SRH in adults over 65 years of age and whether this relationship differs by sex using data from the Korean Community Health Survey (CHS) 2018.

## MATERIALS AND METHODS

### Data collection and participants

The CHS is a nationwide survey conducted by the Korea Disease Control and Prevention Agency under the provisions of the Community Health Act [20]. The survey aims to produce health statistics for the purpose of planning and evaluating community healthcare plans. The target population for this survey was adults aged over 19 years residing in dwelling units served by 254 community health centers. The survey subjects were selected using a complex stratified, multistage probability cluster sampling.

This study utilized data from the 2018 CHS, which directly measured participants’ heights and weights to account for the limitations of older adults, since self-reported BMI may underestimate actual BMI [21]. Skilled investigators conducted one-to-one computerassisted personal interviews and anthropometric surveys between August 15, 2018 and October 31, 2018, in selected households. The study population consisted of 72,475 adults aged over 65 years, out of a total of 220,890 participants, with 59,628 people finally included after excluding 12,804 individuals who refused or were unable to provide anthropometric measurements and 43 people who did not respond to the SBI question. Of the 43 individuals excluded due to the inability to measure their BMI, 23 persons also did not respond to the SBI question.

### Variables

In this study, the dependent variable SRH was assessed through the question, “How do you usually assess your health?” The answers to that question were categorized into 5 levels: very good, good, fair, bad, and very bad. Consistent with previous research, responses were dichotomized into poor (bad+ very bad) and not poor (very good+ good+ fair) categories [6,15].

BMI was calculated by dividing the measured weight (kg) by the squared value of the measured height (m^2^). Heights and weights were obtained to the first decimal place using an automated measuring system, and participants wore only a single layer of thin clothing during the measurements. Obesity was classified according to Korean BMI standards [22], with BMI values < 18.5 kg/m^2^ categorized as underweight, 18.5-22.9 kg/m^2^ as normal, 23.0-24.9 kg/m^2^ as overweight, 25.0-29.9 kg/m^2^ as stage 1 obesity, and ≥ 30.0 kg/m^2^ as stage 2 obesity.

To evaluate SBI, participants were asked the question, “What do you think of your current body shape?” Responses were categorized as “much too thin,” “a bit thin,” “exactly the right weight,” “a bit fat,” or “much too fat,” and this question was similar to those used in prior studies [20,21].

Additionally, age, education level, level of stress, lifetime smoking, lifetime alcohol drinking, exercise, marital status, depression, hypertension, and diabetes mellitus were selected as potential confounding variables that could impact SRH based on previous research findings [4-6].

### Statistical analysis

The differences among variables according to the levels of the SRH were analyzed using the complex-sample t-test or the complex-sample Rao-Scott chi-square test because the CHS has a complex sampling design [23]. Given that the evaluation of SRH differs between men and women [24], we conducted separate analyses for men and women.

For examining whether non-linear associations exist between BMI and SRH, a restricted cubic spline was constructed with 4 knots at the 5th, 25th, 75th, and 95th percentiles of measured BMI using the R package “rms” (https://CRAN.R-project.org/package=rms) [25]. We used a standard value of 23.0 kg/m^2^ for measured BMI as the reference value against which odds ratios (ORs) for restricted cubic plots for each 1-kg/m² unit of BMI were calculated. We conducted two restricted cubic splines. The first model included age, education, subjective stress, lifetime smoking, lifetime alcohol drinking, exercise, marital status, depression, hypertension, and diabetes mellitus as covariates. The other added 5 categories of SBI to the first model.

We estimated the ORs and 95% confidence intervals (CIs) using the R package “survey” (https://CRAN.R-project.org/package=survey) to investigate the relationships among BMI, SBI, and SRH because the CHS has a complex sampling design [23]. Adjusted model 1 included age, education, subjective stress, lifetime smoking, lifetime alcohol drinking, exercise, marital status, depression, hypertension, and diabetes mellitus as confounding variables. Adjusted model 2 added SBI or BMI to the analyses. Multi-collinearity was not confirmed in logistic models (all variance inflation factors were < 5). We also examined the interaction between BMI and SBI.

All statistical analyses were conducted using R version 4.0.3 (R Foundation for Statistical Computing, Vienna, Austria). The significance level was set at p< 0.05.

### Ethics statement

The Institutional Review Board of the Chungnam National University Hospital (No. 2022-08-036) approved this study.

## RESULTS

### Subject characteristics

Table 1 presents data on the relationships among sex, SRH, and various demographic and health-related factors. Older age, underweight, stage 2 obesity, and perceiving oneself as too thin or too fat were significantly associated with poorer SRH. Additionally, lower education, higher levels of subjective stress, ever smoking in one’s lifetime, never drinking alcohol in one’s lifetime, less exercise, never marrying (for men), being widowed (for women), having depression, having hypertension, and having diabetes mellitus were significantly associated with poorer SRH. These associations were similar for both men and women, but poor SRH was more prevalent in women.

### Prevalence of poor self-rated health according to measured body mass index and self-perceived body image

**Figure 1.**
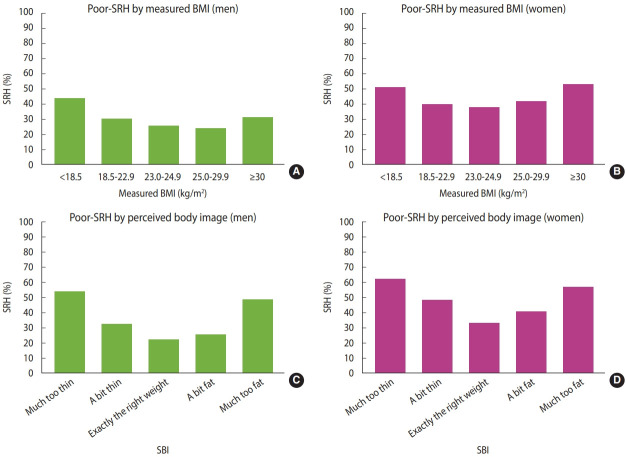
Prevalence of poor SRH according to measured BMI (A: men, B: women) and SBI (C: men, D: women) in aged over 65 years. Proportions were expressed as weighted proportions. SRH, self-rated health; BMI, body mass index; SBI, self-perceived body image.

The prevalence of poor SRH showed a reverse J-shaped relationship for men and a U-shaped relationship for women; it was most frequent in underweight men while in women with stage 2 obesity. According to the SBI, the prevalence of poor SRH showed a U-shape relationship for both men and women; it was the least frequent in those who perceived themselves as exactly the right weight (Table 1 and Figure 1). Stratified analyses of the prevalence of poor SRH using the SBI category for the association between BMI and SRH in the weighted sample are presented in Supplementary Material 1.

### Non-linear relationship between body mass index and self-rated health considering self-perceived body image and sex

**Figure 2.**
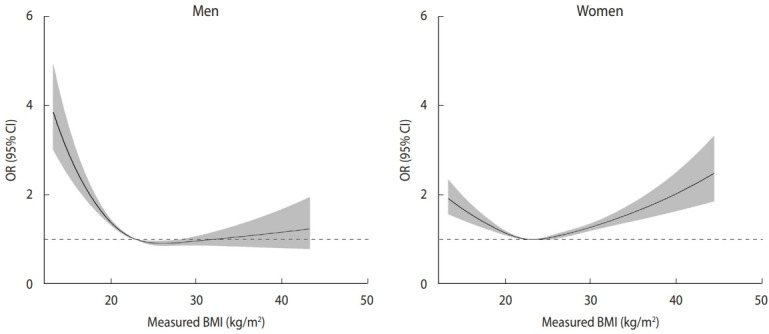
Non-linear relationships using restricted cubic splines between BMI and SRH in men (A) and women (B) aged over 65 years. Restricted cubic splines were used to model the non-linear relationship between BMI and poor SRH using 4 knots at pre-specified locations according to the percentiles of the BMI distribution (i.e., the 5th, 25th, 75th, and 95th percentiles). Curves show ORs compared with the reference BMI of 23 kg/m^2^. The gray band represents the 95% CI. This model was adjusted for age, education level, subjective stress, lifetime smoking, lifetime alcohol drinking, exercise, marital status, depression, hypertension, and diabetes mellitus. BMI, body mass index; SRH, self-rated health; OR, odds ratio; CI, confidence interval.

**Figure 3.**
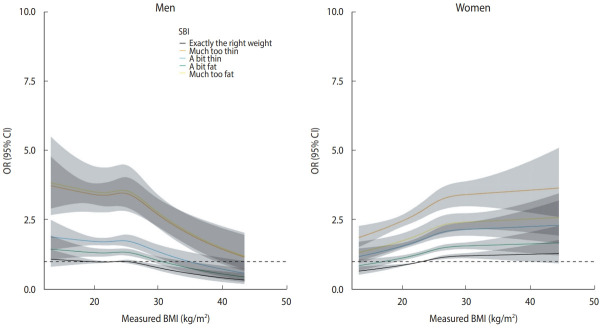
Non-linear relationships using restricted cubic splines between BMI and SRH in men (A) and women (B) aged over 65 years after adjusting for SBI. Restricted cubic splines were used to model the non-linear relationship between BMI and poor SRH using 4 knots at pre-specified locations according to the percentiles of the BMI distribution (i.e., the 5th, 25th, 75th, and 95th percentiles). Curves show ORs compared with the reference BMI of 23 kg/m^2^. The gray band represents the 95% CI. This model was adjusted for age, education level, subjective stress, lifetime smoking, lifetime alcohol drinking, exercise, marital status, depression, hypertension, diabetes mellitus, and SBI. BMI, body mass index; SRH, self-rated health; OR, odds ratio; CI, confidence interval; SBI, self-perceived body image.

Figure 2 presents restricted cubic splines depicting the change in OR for poor SRH with reference to a BMI of 23.0 kg/m^2^, adjusted confounding variables. In men, the curve showed a reverse J-shape (underweight and normal weight increased risk of poor SRH), while in women, the curve showed a U-shape or J-shape. However, including SBI in the model eliminated these relationships and resulted in an inverted U-shape with a negative direction as the obesity stage increased in men and a nearly linear positive relationship in women (Figure 3).

Figure 3 illustrates that among men with the same BMI, those whose SBI was not in the “exactly the right weight” category had a higher risk for poor SRH. Interestingly, this effect was similar for those who thought they were much too fat and those who thought they were much too thin. Additionally, for individuals with the same SBIs, the risk for poor SRH was highest at a BMI of 25.0 kg/m^2^. Among men whose SBI was “exactly the right weight,” a BMI over approximately 28.0 kg/m^2^ appeared to decrease the risk of poor SRH.

Similarly, even among women with the same BMI, those whose SBI was not in the “exactly the right weight” category had a higher risk for poor SRH. In contrast to men, women showed the highest risk for poor SRH when they thought they were much too thin. Moreover, for women with the same SBI, higher BMI values were associated with higher ORs for poor SRH. However, among women whose SBI was “exactly the right weight” and who had a normal or underweight BMI, the risk of poor SRH was lower.

### Relationships among body mass index, self-perceived body image, and self-rated health

There were no statistically significant interactions between BMI and SBI for both men and women. In the crude model, underweight men showed a significant association with poor SRH, but this association did not remain significant in adjusted model 2. Men who were overweight and in stage 1 obesity had a lower risk of poor SRH than men with normal weight in the crude model, but these relationships were not significant in adjusted model 2. For women, the association between BMI and poor SRH displayed a U-shaped pattern in the crude model, indicating that both underweight and higher BMI categories were associated with poorer SRH. However, in adjusted model 2, the association disappeared. In adjusted model 2, overweight (OR, 1.11; 95% CI, 1.01 to 1.23) and stage 2 obesity (OR, 1.24; 95% CI, 1.04 to 1.47) were related to a higher risk of poor SRH in women (Table 2).

Meanwhile, the risk of poor SRH according to SBI showed a U-shape for both men and women in adjusted model 2. The greatest increase in risk was observed in the “much too fat” category of SBI for men (OR, 3.78; 95% CI, 2.74 to 5.20) and in the “much too thin” category for women (OR, 2.60; 95% CI, 2.25 to 3.01). The second-highest risk for poor SRH was observed in the “much too thin” category for men (OR, 3.42; 95% CI, 2.82 to 4.14) and the “much too fat” category for women (OR, 2.26; 95% CI, 1.88 to 2.72). The ORs of BMI categories for poor SRH stratified by the SBI category in the weighted sample are presented in Supplementary Material 2.

## DISCUSSION

The relationship between BMI and poor SRH in men and women aged over 65 years is complex, with a reverse J-shaped or J-shaped relationship. However, including SBI in the model eliminated these relationships. Instead, among men, the relationship changed to an inverted U-shape with a negative direction with the highest risk of poor SRH observed in the underweight to overweight range, while for women, a nearly linear positive relationship was observed. Notably, even among people with the same BMI, those who perceived their weight as not “exactly the right weight” had a higher risk of poor SRH than those who perceived their weight as “exactly the right weight” in both men and women. Older men who thought they were much too fat or too thin had similar highest risks of poor SRH, whereas older women who thought they were too thin had the highest risk of poor SRH. Therefore, both BMI and SBI are related to SRH in those aged over 65 years, but in different ways according to sex.

The study found that the lowest risk for poor SRH occurred when the SBI was “exactly the right weight,” indicating that SBI is associated with SRH independent of BMI. These results are consistent with previous studies conducted on younger adults, middle-aged adults, or all adults [16,17]. The study also showed that most older adults who perceived their weight as not “exactly the right weight” had a high risk of poor SRH, regardless of their BMI. Thus, it is important to consider SBI and educate people about their accurate weight status as evaluated by BMI when planning and implementing interventions. Just like younger or middle-aged adults, interventions aimed at improving weight-related lifestyle habits in older adults should consider SBI and provide education about accurate weight status. Meanwhile, a reverse interpretation of the results suggests that since many obese older adults consider themselves to have a normal weight, educating them about the obesity criteria as defined by BMI could reduce the risk of poor SRH [19].

This study found that older women who perceived themselves as thin had a higher risk of poor SRH than those who thought they were fat. These results are consistent with previous studies [16,17] and may be related to the “obesity paradox,” which suggests that the risk of mortality is lower in older adults who are overweight or in an early stage of obesity [26].

In contrast, there was no difference in the risk of poor SRH between men who perceived themselves as very thin and those who thought they were very fat. The differences in risk perception between men and women may be due to differences in their assessments of SRH. Men tend to evaluate SRH in terms of life-threatening diseases, while women consider both life-threatening and non-life-threatening diseases, as well as other health-related or non-health-related factors [23].

When we considered both BMI and SBI together in the model and examined their relationship with poor SRH, we found sex differences. Among women, higher BMI was associated with a greater risk of poor SRH, regardless of their SBI. This means that even if some women perceived themselves as thin, their risk of poor SRH increased with increasing BMI. Additionally, for women who thought they were fat, their SRH worsened as their BMI increased. These findings suggest that an increase in BMI in older women is associated with poor SRH, regardless of their SBI status. Several studies have demonstrated that an increase in BMI is associated with a higher prevalence of chronic diseases, lower quality of life, increased risk of depression, arthritis, and decreased physical function [7-11]. Our study findings support these previous reports by showing that an increase in BMI was linked to a higher risk of poor SRH in older women, regardless of their SBI status. Thus, the negative health effects of obesity in women may be related, in part, to the association between increased BMI and poor SRH.

The study found that in older men, the relationship between BMI, SBI, and poor SRH differed from that of women. In men, the risk for poor SRH maintained high up to the overweight stage, but beyond that point, the risk for poor SRH decreased with increasing BMI. This pattern was particularly evident in men who considered themselves very thin or very fat. This suggests that in men, SBI may be a stronger predictor of poor SRH than BMI. Furthermore, while the model not including the SBI suggested that low weight was closely related to poor SRH in men, this relationship decreased when SBI was included in the model. Given that people often have incorrect perceptions of their BMI, the study suggests encouraging elderly people, especially men, to measure their weight regularly and pay attention to weight control through a healthy diet and exercise. This could help older men maintain a healthy SBI and BMI and improve their SRH.

Logistic regression analyses revealed no significant interaction between BMI and SBI for poor SRH. This implies that we should consider BMI and SBI independently to assess their effects on SRH. In men, the risk of poor SRH decreased as BMI increased in the adjusted model. However, these associations were not statistically significant. In women, higher BMI was associated with an increased risk of poor SRH. Nevertheless, in the restricted cubic splines analysis, after adding SBI to the model, statistically significant associations between BMI and SRH showed a non-linear pattern. The authors hypothesized that differences exist between logistic regression analysis and restricted cubic splines analysis. Logistic regression analysis compares the association between categorical independent variables and dependent variables, while restricted cubic splines analysis uses a reference of a single point value. We considered that restricted cubic splines analysis reflects the real association more accurately than logistic regression analysis, since the association is non-linear. There may be a stronger tendency among older men to associate a high BMI, indicating a robust build, with good health and a low BMI, indicating a slender build, with poor health. Instead, older women could perceive a high BMI as bad for their health. Therefore, referencing a BMI of 23.0 kg/m^2^ could more prominently show this phenomenon than using a categorical reference.

This study has several limitations. First, because it was crosssectional, we could not establish causality among BMI, SBI, and SRH. Second, unlike some prior studies [12,13,28], we did not collect data on recent weight loss, changes in SRH over time, or changes in BMI. Third, all participants were older Korean adults, and the generalizability of our findings to other cultures is uncertain. It should be noted that other studies have found that the association between obesity and SRH varies across countries [29,30].

Despite these limitations, this study is significant for several reasons. First, it was conducted in the Korea, where aging is progressing at a rapid pace due to the country’s rapid economic development after the Korean War [31]. Second, the study used a nationally representative sample selected through complex stratified, multistage probability cluster sampling, making the findings more generalizable to other countries with similar environments.

Moreover, this study used restricted cubic splines to analyze the non-linear relationships among BMI, SBI, and SRH. This method takes advantage of the characteristics of BMI as a continuous variable, enabling a better understanding of the complex relationship between these variables in older adults. This approach may be beneficial in other studies investigating similar relationships.

The findings of this study emphasize the importance of considering sex and body image perceptions when assessing the relationship between BMI and SRH in older adults, especially in men. This information can help in developing effective strategies for obesity counseling for older adults, including an evaluation of SBI and education based on measured BMI.

## Figures and Tables

**Figure f4-epih-45-e2023061:**
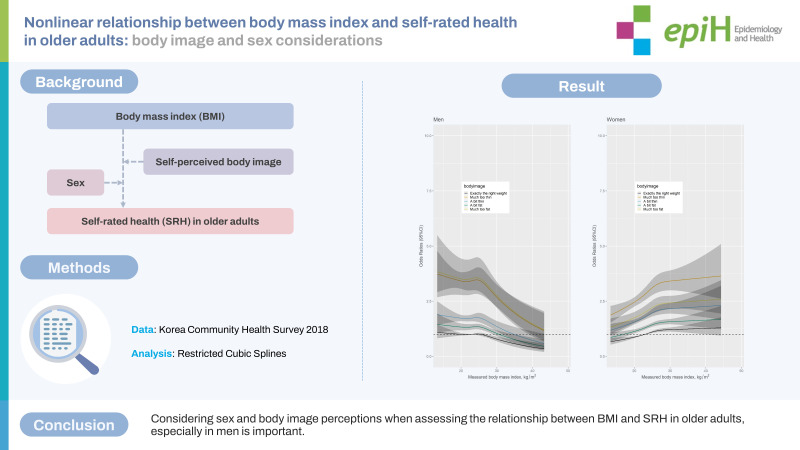


**Table 1.** General characteristics of the subjects according to SRH (n=59,628)^1^

Characteristics		Men			Women		
		Not poor SRH (n=16,858)	Poor SRH (n=7,244)	p-value^2^	Not poor SRH (n=19,637)	Poor SRH (n=15,889)	p-value^2^
Age, mean±SE (yr)		72.8±0.0	75.0±0.1	<0.001	73.1±0.1	75.4±0.1	<0.001
BMI (kg/m^2^)				<0.001			<0.001
	<18.5	643 (56.7)	666 (43.3)		572 (49.4)	763 (50.7)	
	18.5-22.9	5723 (70.7)	6435 (29.3)		2760 (60.6)	5109 (39.4)	
	23.0-24.9	4690 (74.6)	4821 (25.4)		1761 (62.8)	3342 (37.2)	
	25.0-29.9	5397 (76.3)	6783 (23.7)		1957 (58.6)	5495 (41.4)	
	≥30.0	405 (69.3)	932 (30.7)		194 (46.8)	1180 (53.2)	
Self-perceived body image				<0.001			<0.001
	Much too thin	871 (46.2)	1,122 (53.8)		1,183 (37.9)	2,116 (62.1)	
	A bit thin	3,316 (68.0)	1,828 (32.0)		3,142 (52.1)	3,377 (48.0)	
	Exactly the right weight	8,857 (78.5)	2,704 (21.5)		9,519 (67.2)	5,530 (32.8)	
	A bit fat	3,586 (74.9)	1,352 (25.1)		5,192 (59.2)	4,015 (40.8)	
	Much too fat	228 (51.4)	238 (48.6)		601 (43.5)	851 (56.5)	
Education				<0.001			<0.001
	None	886 (56.4)	754 (43.6)		4,730 (44.1)	6,162 (55.9)	
	Elementary school	5,581 (65.1)	3,141 (34.9)		9,395 (57.0)	7,390 (43.0)	
	Middle school	3,673 (71.3)	1,487 (28.8)		2,650 (65.8)	1,430 (34.2)	
	High school	4,249 (78.4)	1,308 (21.6)		2,112 (75.4)	724 (24.6)	
	College or over	2,449 (82.2)	547 (17.8)		726 (81.9)	169 (18.1)	
Subjective stress				<0.001			<0.001
	Very much	175 (42.1)	298 (57.9)		315 (32.3)	836 (67.7)	
	Much	1,376 (52.7)	1,378 (47.3)		2,232 (41.0)	3,690 (59.0)	
	A little	7,386 (75.5)	2,914 (24.5)		8,505 (61.7)	6,408 (38.3)	
	None	7,912 (77.7)	2,634 (22.4)		8,569 (67.6)	4,901 (32.4)	
Lifetime smoking				<0.001			<0.001
	Yes	12,855 (71.9)	5,794 (28.1)		626 (45.6)	799 (54.5)	
	No	4,001 (76.2)	1,450 (23.8)		19,011 (59.8)	15,090 (40.2)	
Lifetime alcohol drinking				<0.001			<0.001
	Yes	14,894 (73.4)	6,257 (26.6)		11,942 (61.4)	8,847 (38.7)	
	No	1,964 (68.7)	987 (31.3)		7,695 (55.8)	7,042 (44.2)	
Walking (day/wk)				<0.001			<0.001
	0	3,251 (57.4)	2,345 (42.6)		3,462 (40.5)	4,907 (59.5)	
	1-3	2,918 (72.0)	1,247 (28.0)		4,051 (55.2)	3,500 (44.9)	
	4-6	3,219 (76.7)	1,071 (23.3)		4,235 (66.5)	2,533 (33.5)	
	7	7,460 (77.3)	2,565 (22.7)		7,857 (65.5)	4,908 (34.5)	
Marital status				<0.001			<0.001
	Married	14,787 (73.8)	6,103 (26.2)		9,605 (62.2)	6,901 (37.8)	
	Widowed	1,307 (68.9)	733 (31.1)		9,426 (55.7)	8,601 (44.3)	
	Divorced or separated	693 (65.0)	351 (35.0)		502 (61.1)	347 (38.9)	
	Never married	63 (56.6)	51 (43.4)		92 (77.2)	36 (22.8)	
Depression				<0.001			<0.001
	Yes	432 (41.3)	653 (58.7)		1,014 (38.0)	1,898 (62.0)	
	No	16,418 (74.7)	6,586 (25.4)		18,614 (61.5)	13,966 (38.5)	
Hypertension				<0.001			<0.001
	Yes	8,052 (68.6)	4,224 (31.4)		10,593 (52.9)	10,672 (47.1)	
	No	8,804 (77.6)	3,017 (22.4)		9,037 (68.1)	5,209 (31.9)	
Diabetes mellitus				<0.001			<0.001
	Yes	3,028 (61.8)	2,111 (38.2)		3,177 (45.8)	4,142 (54.2)	
	No	13,828 (76.1)	5,128 (23.9)		16,450 (63.0)	11,738 (37.0)	

Values are presented as number (weighted %). SRH, self-rated health; SE, standard error; BMI, body mass index.

1 Not poor SRH means very good, good, or fair SRH; Poor SRH means bad or very bad SRH.

2 By the complex-sample t-test or the complex-sample Rao-Scott chi-square test.

**Table 2.** Relationships^1^ among BMI, SBI, and SRH in both men and women over 65 years of age^2^

Variables		Men			Women		
		Crude	Model 1	Model 2	Crude	Model 1	Model 2
BMI (kg/m^2^)							
	<18.5	1.84 (1.54, 2.21)	1.66 (1.36, 2.03)	1.01 (0.81, 1.26)	1.58 (1.33, 1.87)	1.49 (1.24, 1.79)	0.99 (0.81, 1.22)
	18.5-22.9	1.00 (reference)	1.00 (reference)	1.00 (reference)	1.00 (reference)	1.00 (reference)	1.00 (reference)
	23.0-24.9	0.82 (0.74, 0.92)	0.86 (0.76, 0.96)	1.13 (0.99, 1.28)	0.91 (0.84, 0.99)	0.94 (0.86, 1.04)	1.11 (1.01, 1.23)
	25.0-29.9	0.75 (0.68, 0.83)	0.73 (0.66, 0.82)	0.87 (0.75, 1.01)	1.09 (1.01, 1.17)	1.01 (0.93, 1.10)	1.10 (0.99, 1.22)
	≥30.0	1.07 (0.82, 1.38)	0.97 (0.73, 1.30)	0.75 (0.53, 1.05)	1.75 (1.53, 2.01)	1.43 (1.24, 1.65)	1.24 (1.04, 1.47)
SBI							
	Much too thin	4.26 (3.67, 4.94)	-	3.42 (2.82, 4.14)	3.35 (2.97, 3.76)	-	2.60 (2.25, 3.01)
	A bit thin	1.72 (1.55, 1.91)	-	1.69 (1.49, 1.92)	1.88 (1.73, 2.06)	-	1.67 (1.50, 1.85)
	Exactly the right weight	1.00 (reference)	-	1.00 (reference)	1.00 (reference)	-	1.00 (reference)
	A bit fat	1.22 (1.10, 1.36)	-	1.37 (1.20, 1.56)	1.41 (1.31, 1.52)	-	1.39 (1.27, 1.53)
	Much too fat	3.45 (2.63, 4.53)	-	3.78 (2.74, 5.20)	2.66 (2.27, 3.11)	-	2.26 (1.88, 2.72)

Values are presented as odds ratio (95% confidence interval) for poor SRH. BMI, body mass index; SBI, self-perceived body image; SRH, self-rated health.

1 By survey logistic regression analyses.

2 Model 1 was adjusted for age, education, subjective stress, lifetime smoking, lifetime alcohol drinking, exercise, marital status, depression, hypertension, and diabetes mellitus; Model 2: model 1+SBI.

## SUPPLEMENTARY MATERIALS

Supplementary Material 1. Stratified analyses of the prevalence of poor SRH using SBI for the association between BMI and SRH (n=59,628).

Supplementary Material 2 Stratified analyses of the risk of poor SRH using SBI for the association between BMI and SRH.
