# Supplementary material for: Non-linear relationship between body mass index and self-rated health in older Korean adults: body image and sex considerations
Source: Epidemiol Health. 2023 Jun 20;45:e2023061. doi: 10.4178/epih.e2023061 (PMC10667579; doi:10.4178/epih.e2023061)
Supplement: Supplementary Material 1. — Stratified analyses of the prevalence of poor SRH using SBI for the association between BMI and SRH (n=59,628). [file epih-45-e2023061-Supplementary-1.docx]

Supplementary Material 1. Stratified analyses of the prevalence of poor SRH using SBI for the association between BMI and SRH (n=59,628).

|  | Prevalence of poor SRH (n [%]) | | | | | | | | | |
| --- | --- | --- | --- | --- | --- | --- | --- | --- | --- | --- |
|  | Men | | | | | Women | | | | |
|  | Much too thin | A bit thin | Exactly the right weight | A bit too fat | Much too fat | Much too thin | A bit thin | Exactly the right weight | A bit too fat | Much too fat |
| P-value | 0.1064 | 0.1657 | 0.5063 | 0.035 | 0.047 | <0.001 | <0.001 | <0.001 | <0.001 | 0.1011 |
| **BMI**  **(kg/m^2^)** |  |  |  |  |  |  |  |  |  |  |
|  |  |  |  |  |  |  |  |  |  |  |
| < 18.5 | 411 (52.2) | 1,37 (31.7) | 16 (17.1) | 3 (64.2) | 5 (82.8) | 516 (54.5) | 199 (48.9) | 37 (24.4) | 9 (95.4) | 2 (39.3) |
| 18.5-22.9 | 635 (54.0) | 1,250 (31.0) | 805 (20.6) | 56 (31.0) | 14 (79.5) | 1,302 (64.3) | 2,033 (44.9) | 1,579 (28.9) | 183 (33.1) | 12 (61.2) |
| 23.0-24.9 | 58 (72.3) | 349 (36.9) | 1,090 (22.4) | 260 (29.0) | 4 (49.0) | 192 (71.9) | 708 (54.0) | 1,792 (32.6) | 634 (37.4) | 16 (44.5) |
| 25.0-29.9 | 15 (45.3) | 91 (32.1) | 779 (21.3) | 943 (23.7) | 129 (47.9) | 88 (70.3) | 416 (57.7) | 1,999 (37.5) | 2,632 (41.2) | 360 (51.6) |
| ≥ 30 | 3 (17.2) | 1 (32.5) | 14 (16.4) | 90 (25.7) | 86 (43.6) | 18 (71.9) | 21 (72.6) | 123 (41.1) | 557 (48.8) | 461 (61.7) |
| **Total** | 1,122 (53.8) | 1,828 (32.0) | 2,704 (21.5) | 1,352 (25.1) | 238 (48.6) | 2,116 (62.1) | 3,377 (48.0) | 5,530 (32.8) | 4,015 (40.8) | 851 (56.5) |

Data were expressed as n (weighted proportion, %).

P-values were obtained by complex sample Rao-Scott chi-square test.

SBI: self-perceived body image; BMI: body mass index; SRH: self-rated health.
