# Supplementary material for: Non-linear relationship between body mass index and self-rated health in older Korean adults: body image and sex considerations
Source: Epidemiol Health. 2023 Jun 20;45:e2023061. doi: 10.4178/epih.e2023061 (PMC10667579; doi:10.4178/epih.e2023061)
Supplement: Supplementary Material 2 — Stratified analyses of the risk of poor SRH using SBI for the association between BMI and SRH. [file epih-45-e2023061-Supplementary-2.docx]

Supplementary Material 2. Stratified analyses of the risk of poor SRH using SBI for the association between BMI and SRH.

|  | Men | | | | | | Women | | | | | |
| --- | --- | --- | --- | --- | --- | --- | --- | --- | --- | --- | --- | --- |
|  | Much too thin | A bit thin | Exactly the right weight | A bit too fat | Much too fat | P_int_ | Much too thin | A bit thin | Exactly the right weight | A bit too fat | Much too fat | P_int_ |
| BMI  (kg/m^2^) |  |  |  |  |  | 0.3103 |  |  |  |  |  | 0.4566 |
| < 18.5 | 1.00  (0.74-1.35) | 0.99  (0.69-1.40) | 0.59  (0.23-1.49) | 3.56  (0.4-31.94) | 0.77  (0.06-9.62) |  | 0.85  (0.65-1.1) | 1.31  (0.91-1.87) | 0.68  (0.32-1.41) | 25.88  (4.72-141.87) | 0.08  (0.01-0.94) |  |
| 18.5-22.9 | Reference | | | | |  | Reference | | | | |  |
| 23.0-24.9 | 1.84  (0.74-4.55) | 1.08  (0.85-1.36) | 1.15  (0.97-1.36) | 1.09  (0.64-1.85) | 0.11  (0.01-0.97)^*^ |  | 1.23  (0.79-1.91) | 1.12  (0.92-1.38) | 1.10  (0.96-1.25) | 1.40  (1.02-1.93) | 0.99  (0.11-8.61) |  |
| 25.0-29.9 | 0.35  (0.09-1.33) | 0.68  (0.44-1.06) | 0.96  (0.81-1.15) | 0.78  (0.47-1.29) | 0.27  (0.07-1.07) |  | 1.07  (0.54-2.13) | 0.98  (0.74-1.3) | 1.14  (0.99-1.31) | 1.29  (0.96-1.73) | 0.76  (0.1-5.71) |  |
| ≥ 30 | 0.18  (0.03-1.15) | 0.85  (0.11-6.83) | 0.47  (0.20-1.10) | 0.76  (0.40-1.42) | 0.28  (0.07-1.13) |  | 0.84  (0.26-2.76) | 1.82  (0.6-5.55) | 1.12  (0.74-1.7) | 1.46  (1.05-2.03)^*^ | 0.83  (0.11-6.2) |  |

Data were expressed as OR (95% confidence interval) for poor SRH.

SBI: self-perceived body image; BMI: body mass index; SRH: self-rated health; OR: odds ratio.

ORs were calculated by survey logistic regression analyses adjusting age, education, subjective stress, lifetime smoking, lifetime alcohol drinking, exercise, marital status, depression, hypertension, and diabetes mellitus.

P_int_ means the P-value of interaction between BMI and SBI.
